# Vitamin D Receptor Gene FOKI Polymorphism Contributes to Increasing the Risk of HIV-Negative Tuberculosis: Evidence from a Meta-Analysis

**DOI:** 10.1371/journal.pone.0140634

**Published:** 2015-10-20

**Authors:** Chun Xu, Peijun Tang, Cheng Ding, Chang Li, Jun Chen, Zhenlei Xu, Yi Mao, Meiying Wu, Jun Zhao

**Affiliations:** 1 Department of Cardiothoracic Surgery, The First Hospital Affiliated to Soochow University, Soochow, Jiangsu, People's Republic of China; 2 Department of Tuberculosis, The Affiliated Infectious Hospital of Soochow University, Soochow, Jiangsu, People's Republic of China; Harbin Medical University, CHINA

## Abstract

**Background:**

Vitamin D receptor (VDR) gene *Fok*I polymorphism have been studied in relation to tuberculosis (TB) in many populations and provided inconsistent results. In this study, we carried out a meta-analysis to derive a more reliable assessment on *Fok*I polymorphism and the risk of HIV-negative TB.

**Methods:**

The Embase, PubMed, and Cochrane Library databases were used to undertake a comprehensive systematic literature review of all current published VDR gene *FOKI* association studies aimed at the risk of TB up to June 30, 2015. Odds ratios (ORs) and the corresponding 95% confidence intervals (CIs) were used to measure the strength of the models.

**Results:**

A total of 14 studies (1,668 cases and 1,893 controls) were retrieved in the meta-analysis. The pooled OR was 1.60 (95% = 1.28–1.97, *P*<0.001; *I* ^*2*^ = 29.5%, and *P* = 0.141 for heterogeneity) in the best genetic model (recessive model: ff vs. fF+FF). In the subgroup analysis by ethnicities, a significantly increased risk was found in the Asian group (OR = 1.82, 95% CI = 1.42–2.33, *P*<0.001; *I* ^*2*^ = 31.0%, and *P* = 0.150 for heterogeneity) in the recessive model. Similarly, significant associations were also found in the polymerase chain reaction-restriction fragment length polymorphism group, high-quality studies, and the population based or hospital based groups. Moderate heterogeneity was found in this study.

**Conclusion:**

Our results suggested that VDR *Fok*I polymorphism contributes to increasing the risk of TB in HIV-negative individuals, especially in the Asian region. Further studies on this topic in other races are expected to be conducted in future.

## Introduction

Tuberculosis (TB) is a global public health problem and remains a great burden throughout the world, although there has been an overall decline in TB incidence and mortality to this date. In 2013, an estimated 9 million people developed TB, and 1.5 million died from the disease, including 360,000 deaths in HIV-positive people. Many countries have high rates of TB and HIV co-infection [1]. A prospective study indicated a TB incidence rate of 6.9/100 persons per year in patients infected with HIV in India [2].

In recent years, there has been a significant improvement in our understanding that vitamin D can influence the pathophysiology and possible prevention of human disease. Vitamin D is now considered to be a key factor of the body’s defence against TB through its action of enhancing macrophage-mediated eradication of *Mycobacterium tuberculosis* [3]. It has been shown that vitamin D deficiency and insufficiency are associated with a higher risk of active TB [4]. The vitamin D receptor (VDR) gene is located in the chromosomal 12q13 region, and there are four important gene polymorphisms (*Fok*I, *Bsm*I, *Apa*I, *Taq*I). The polymorphisms of *Fok*I of the VDR gene, which transition C to T (rs10735810, usually “F” represented C and “f” for T) at the first of the two potential translation initiation sites in exon 2, is related to plasma vitamin D levels in TB patients [5]. Therefore, the polymorphisms of *Fok*I have been studied in relation to TB in many populations [6–19].

However, previous literature about the associations between *Fok*I polymorphism and the risk of TB has provided inconsistent results [6–19]. A previous meta-analysis found that *Fok*I polymorphism was associated with TB risk with significant heterogeneity [20, 21]. However, they did not stratify by HIV status. Since TB is the frequent major opportunistic infection in HIV-infected patients, genetic susceptibility to TB in HIV patients might also change [22]. We hypothesise that HIV infection status is the source of heterogeneity in previous studies, and that it is necessary to exclude the studies with HIV-positive TB to avoid selection bias, and that the stratification of HIV status would further reveal the relationship between *Fok*I polymorphism and TB. Therefore, a meta-analysis was carried out to derive a more reliable assessment on VDR *Fok*I polymorphism and the risk of HIV-negative TB.

## Methods

This meta-analysis was performed according to the Preferred Reporting Items for Systematic Reviews and Meta-analyses (PRISMA) statement(S1 PRISMA Checklist and S1 Table), including the search strategy, selection criteria, data extraction, and data analysis [23].

### Literature Search

We used the Embase, PubMed, and Cochrane Library databases to undertake a comprehensive systematic literature review of all current published VDR gene *FOKI* association studies aimed at the risk of TB up to June 30, 2015. The search terms were used as follows: *vitamin D receptor* or *VDR* in combination with *polymorphism*, *polymorphisms*, and *mutation* or *variant* in combination with *tuberculosis* or *TB* or *phthisic* or *phthisis*. Two investigators (CX and PT) conducted an extensive literature search independently for all publications. Articles in reference lists were also hand-searched. Only English articles and human studies were searched.

### Inclusion and Exclusion Criteria

Studies aiming to evaluate the association between VDR gene *FOKI* polymorphism and the susceptibility to HIV negative-TB were selected. The inclusion criteria were as follows:

1. Case-control or cohort design studies had to include data regarding the baseline characteristics of the patients (number, age, sex). In addition, the inclusion and exclusion criteria for recruiting TB patients had to be clearly indicated.
2. All the patients in studies had to be HIV-negative.
3. Studies had to offer the ability to extract data for calculating the odds ratio (OR), 95% confidence intervals (CIs), and Hardy-Weinberg equilibrium (HWE).
4. DNA genotyping methods and the sources of cases and controls were stated in studies.

Review articles, case reports, editorials, conference abstracts, letters and family-based studies were excluded.

### Data Extraction

Two reviewers (CX and PT) independently assessed publications for inclusion in the review. Data extracted from eligible studies included the baseline characteristics, such as the first author’s name, publication year, country, ethnicity, total sample size, genotyping method, and source of control group. Details of TB types and genotype frequencies of cases and controls were obtained. HWE was calculated from genotype frequencies of controls. Investigators would try to contact the author to get the original data if the literature could not provide sufficient data. To determine the accuracy of the extracted information by the two investigators, they checked their data if there was a dispute at first. If the two investigators could not reach an agreement, discrepancies were then resolved through discussion by the review team.

### Assessment of Study Quality

To assess the validity of each study, we applied the criteria predefined by Thakkinstian et al. [24], with some criteria modified (Table 1). The following important criteria were assessed: the sources of cases and controls, the total sample size, the specimens of cases, and the Hardy-Weinberg Equilibrium (HWE) of controls. According to the validity criteria shown in Table 1, a study scoring >10 was considered a high-quality study, while a score of ≤10 was classified as a low-quality study; the lowest score was 0 and the highest score was 15 [25].

**Table 1:** Scale for quality assessment.

Criteria	Score
Representativeness of cases	
Selected from population or tuberculosis registry	3
Selected from hospital	2
Not described	1
Representativeness of controls	
Population-based	3
Blood donors or volunteers	2
Hospital-based(cancer-free patients)	1
Not described	0
Specimens of cases determining genotypes	
White blood cells or normal tissues	3
Tumor tissues or exfoliated cells of tissue	0
Hardy-Weinberg equilibrium in controls	
Hardy-Weinberg equilibrium	3
Hardy-Weinberg disequilibrium	0
Total sample size	
≥1000	3
≥500 but <1000	2
≥200 but <500	1
<200	0

### Statistical Analysis

According to a previous meta-analysis [21], f is the risk allele; therefore, the comparison models to access the association between VDR gene *FOKI* polymorphism and the susceptibility to HIV-negative TB including an allelic model (f vs. F), co-dominant model (ff vs. FF, fF vs. FF and ff vs. fF), a dominant model (ff+fF vs. FF), and a recessive model (ff vs. fF+FF). Unadjusted odds ratios (ORs) and the corresponding 95% confidence intervals (CIs) were used to measure the strength of the models because it is difficult to get the all original data from authors.

To dictate the best genetic model and avoid the problem of multiple comparisons, we applied the method for meta-analysis of molecular association studies [26]. OR_1_, OR_2_, and OR_3_ were calculated for the genotypes ff vs. FF (OR_1_), fF vs. FF (OR_2_), and ff vs. fF (OR_3_). These pairwise differences can be used to indicate the best genetic model, as outlined below:

1. If OR_1_ = OR_3_≠1 and OR_2_ = 1(OR_1_ and OR_3_ were equal and had significant effects while OR2 was not significant.), then a recessive model is suggested.
2. If OR_1_ = OR_2_≠1 and OR_3_ = 1, then a dominant model is suggested.
3. If OR_2_ = 1/OR_3_≠1 and OR_1_ = 1, then a complete overdominant model is suggested.
4. If OR_1_ >OR_2_>1 and OR_1_ >OR_3_>1 (or OR_1_<OR_2_<1 and OR_1_ <OR_3_<1), then a dominant model is suggested.

Heterogeneity was assessed by a chi-squared Q test and I-squared (*I* ^*2*^) statistics and classified as low (*I* ^*2*^<25%), moderate (*I* ^*2*^ = 25–50%), and high (*I* ^*2*^>50%), with a cut-off P-value of 0.10. A random-effects model was conducted using the DerSimonian and Laird method to calculate the summary OR and the corresponding 95% CI; otherwise, a fixed-effects model (the Mantel—Haenszel method) was used [27, 28]. The HWE in the controls was tested by the chi-square test for goodness of fit, and a *P*-value <0.05 was considered out of HWE. We also carried out a subgroup analysis by ethnicity, genotyping method, source of controls, TB type, HWE, and score by quality assessment, respectively.

To verify the robustness of the findings, sensitivity analysis was conducted to examine such influences by removing studies one by one, especially removing the study out of HWE, and recalculating the pooled OR and 95% CI. If the corresponding pooled ORs were not qualitatively altered, we considered the results robust. The potential for publication bias was assessed with Begg’s funnel plot and Egger’s test [29, 30].

All the tests in this meta-analysis were conducted with the STATA software (version 12.0; Stata Corporation, College Station, Texas, USA) and RevMan 5.3 (Cochrane Collaboration). A P-value <0.05 was considered statistically significant.

## Results

### Study Inclusion and Characteristics

**Fig 1:**
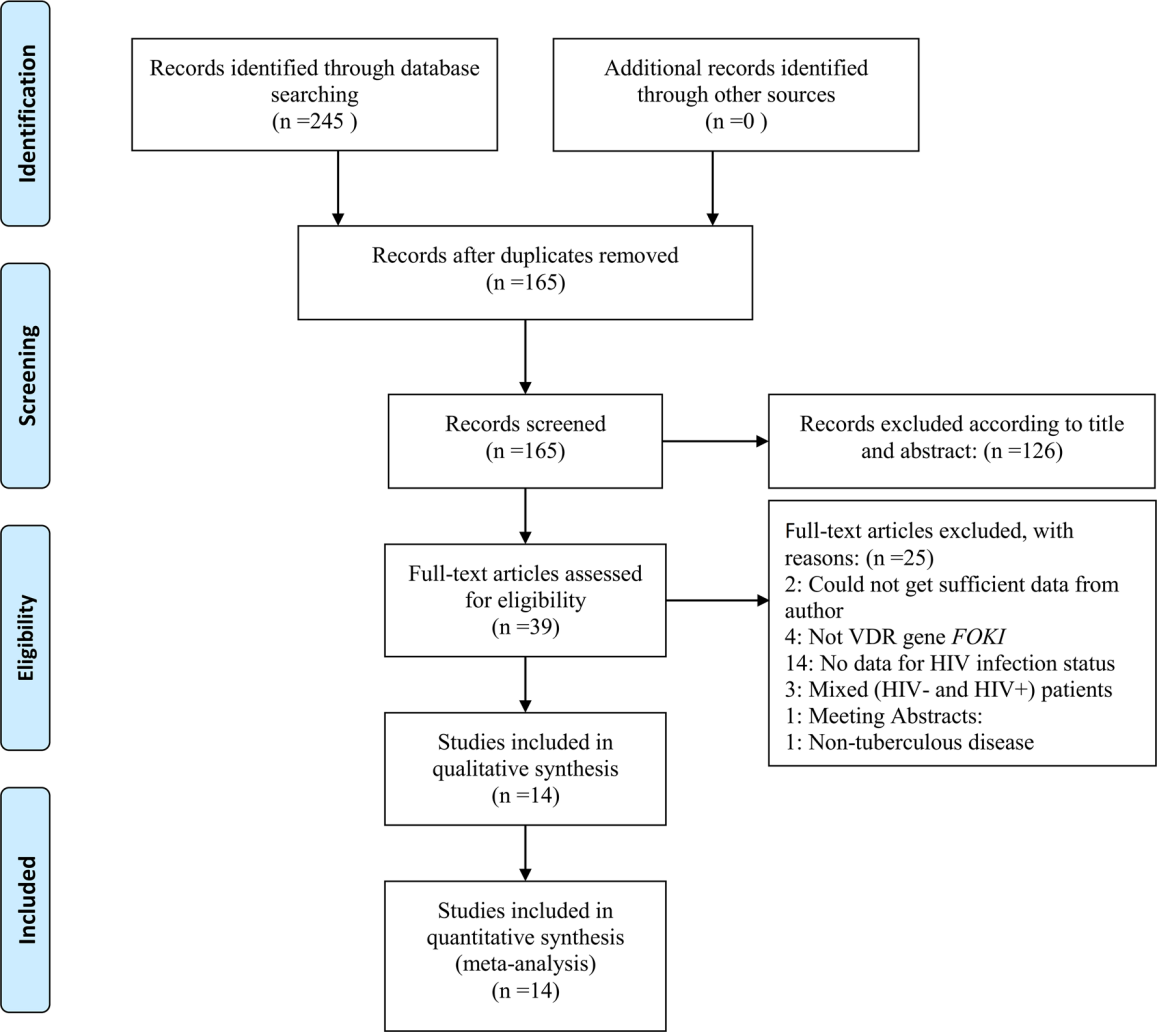
Flow diagram of included studies for this meta-analysis.

The search strategy identified 165 citations. Thirty-nine articles that were thought to be potentially eligible for inclusion were retrieved and evaluated after the titles and abstracts were reviewed, and 25 articles were excluded after full texts were reviewed according to the inclusion and exclusion criteria (Fig 1, S2 Table). Finally, a total of 14 studies, amounting to 1,668 patients and 1,893 control subjects, were retrieved in the meta-analysis [6–19].

Table 2 lists the characteristics of eligible and included studies, including source of control, genotyping method, frequencies of genotype in case and control groups, HWE, and quality score. Of the remaining 14 articles we identified, 11 case-control studies were conducted in the Asian region [6, 8, 9, 12–19], 2 were African [7, 10], and only one was South American [11]. In addition, 11 studies focused on pulmonary TB (PTB), one on spinal TB, one on PTB and meningeal tuberculosis (MTB) and one did not show the type of TB (Table 2). Only one study was out of HWE; it was also considered a low-quality study [14].

**Table 2:** Characteristics of studies included in the meta-analysis.

Study	Year	Country	Total sample		TB	Source of Control	Genotyping method	Cases sequence			Control sequence			*P*_HWE_	Quality scores
			Cases	Control				F/F	F/f	f/f	F/F	F/f	f/f		
Alagarasu	2009	India	105	144	PTB	PB	SSP-PCR	65	31	9	81	59	4	0.076	13
Bobb	2007	South Africa	352	249	PTB	PB	RFLP-PCR	203	129	20	132	104	13	0.192	13
Banoei	2010	Iranian	60	62	PTB	PB	SSP-PCR	30	21	9	29	27	6	0.937	11
Liu	2004	China	120	240	PTB	PB	RFLP-PCR	29	63	28	85	120	35	0.482	12
Lombard	2006	South Africa	104	117	PTB,MTB	HB	SSP-PCR	68	33	3	90	24	3	0.373	11
Roth	2004	Peru	100	100	PTB	HB	RFLP-PCR	9	32	59	7	36	57	0.689	11
SALIMI	2015	Iran	120	131	PTB	HB	RFLP-PCR	65	44	11	93	31	7	0.054	12
Selvaraj	2008	India	51	60	PTB	PB	RFLP-PCR	31	16	4	27	33	0	0.003	8
Selvaraj	2009	India	65	60	PTB	PB	RFLP-PCR	33	29	3	33	26	1	0.102	11
Sinaga	2014	Indonesia	76	76	PTB	HB	RFLP-PCR	27	42	7	30	34	12	0.650	11
Singh	2011	East india	101	225	PTB	HB	RFLP-PCR	55	40	6	96	110	19	0.107	11
Wilkinson	2000	UK	91	116	TB	HB	RFLP-PCR	52	31	8	74	39	3	0.418	11
Wu	2013	China	213	211	PTB	HB	RFLP-PCR	72	96	45	101	88	22	0.664	11
Zhang	2010	China	110	102	spinal-TB	HB	RFLP-PCR	16	43	51	26	47	29	0.433	11

PB, Population—based; HB, Hospital—based; PTB, pulmonary tuberculosis; MTB, meningeal tuberculosis; SSP-PCR, sequence specific primer-polymerase chain reaction; RFLP-PCR, polymerase chain reaction-restriction fragment length polymorphism; HWE, Hardy—Weinberg equilibrium in control population

### Quantitative Data Synthesis

The estimated OR1, OR2, and OR3 were 1.69 (95% CI = 1.19–2.41), 1.06 (95% CI = 0.83–1.35), and 1.48 (95% CI = 1.13–1.95), respectively (Table 3). These indicated that OR_1_ = OR_3_≠1 and OR_2_ = 1, and suggested the genetic model was most likely to be recessive. Therefore, the FF and fF genotypes were combined and compared with ff (ff vs. FF+fF). The pooled OR was 1.60 (95% = 1.28–1.97, P<0.001; *I* ^*2*^ = 29.5%, and *P* = 0.141 for heterogeneity). Summary results of comparisons are listed in Table 3.

**Table 3:** Meta-analysis of *FOKI* Polymorphism and HIV-negative TB risk.

Comparison	Group	No. of studies	Test of association			Heterogeneity	
			OR	95%CI	*P*	I2%	*P*
**ff vs. FF**	overall	14	1.69	1.19–2.41	0.004	39.5	0.064
**fF vs. FF**	overall	14	1.06	0.83–1.35	0.623	57.8	0.004
**ff vs. fF**	overall	14	1.48	1.13–1.95	0.005	19.6	0.240
**ff vs. FF+fF**							
overall		14	1.60	1.28–1.97	<0.001	29.5	0.141
	Asian	11	1.82	1.42–2.33	<0.001	31.0	0.150
	Africa	2	1.10	0.57–2.12	0.778	0.0	0.974
SSP-PCR		3	1.96	0.97–4.00	0.061	0.0	0.537
RFLP-PCR		11	1.56	1.25–1.96	<0.001	40.6	0.078
PTB		11	1.48	1.16–1.89	0.001	34.4	0.123
HWE		13	1.56	1.27–1.92	<0.001	28.1	0.161
Score > 10		13	1.56	1.27–1.92	<0.001	28.1	0.161
	PB	6	1.75	1.22–2.56	0.003	0.0	0.486
	HB	8	1.52	1.16–1.89	0.002	49.1	0.056

PB, Population—based; HB, Hospital—based; PTB, pulmonary tuberculosis; MTB, meningeal tuberculosis; SSP-PCR, sequence specific primer-polymerase chain reaction; RFLP-PCR, polymerase chain reaction-restriction fragment length polymorphism; HWE, Hardy—Weinberg equilibrium in control population

**Fig 2:**
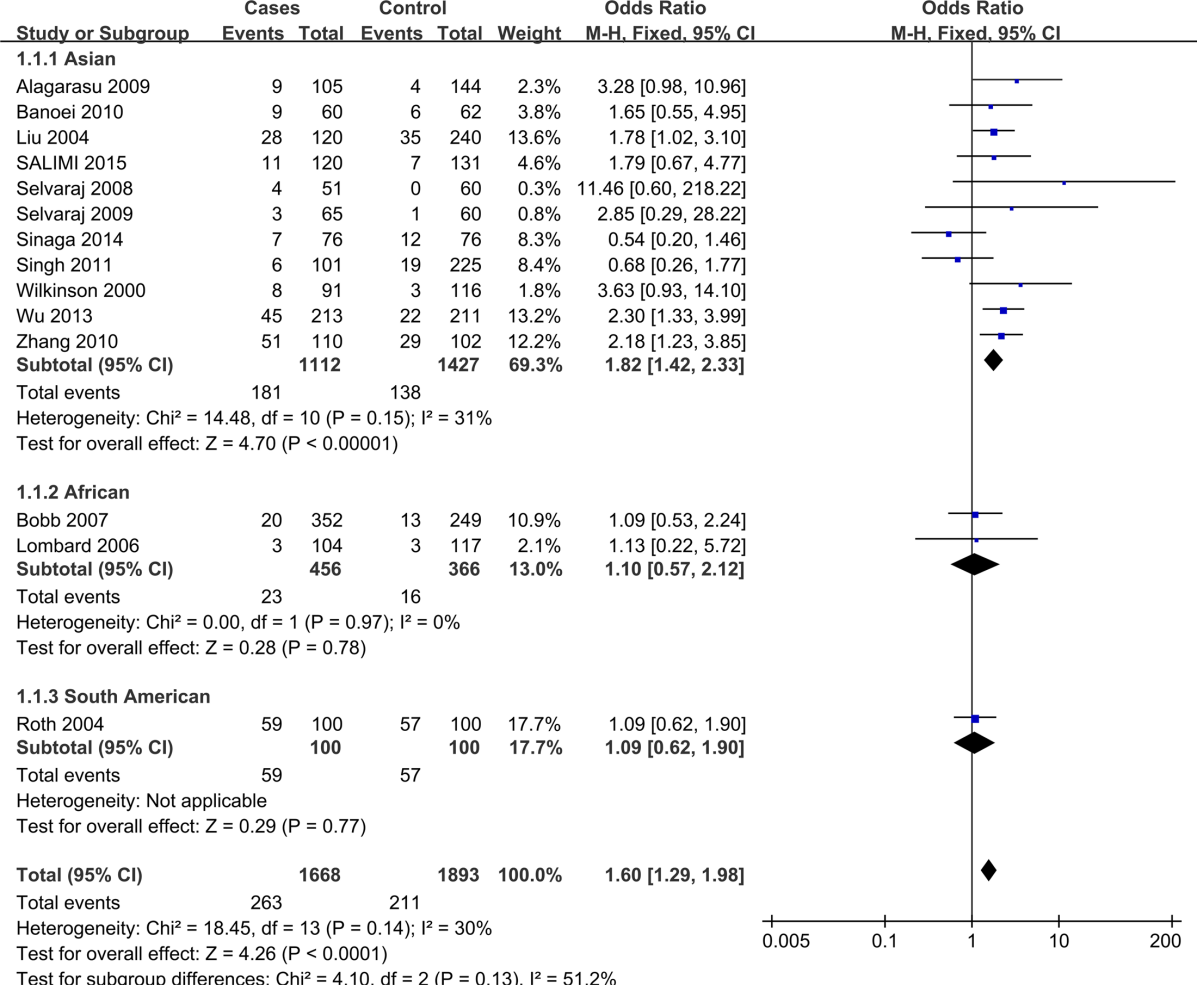
Forest plot of VDR *Fok*I Polymorphism and HIV-negative TB risk in recessive model.

In the subgroup analysis by ethnicities, as shown in Fig 2 and Table 3, a significantly increased risk was found in the Asian group (OR = 1.82, 95% CI = 1.42–2.33, P<0.001; *I* ^*2*^ = 31.0%, and *P* = 0.150 for heterogeneity) in the recessive model. However, no significant associations were found in the African group (OR = 1.10, 95% CI = 0.51–2.13, P = 0.778; *I* ^*2*^ = 0.0%, and *P* = 0.974 for heterogeneity). Because only one study was conducted in the South American population, the heterogeneity and pooled ORs could not be calculated. Significant associations were also found in the polymerase chain reaction-restriction fragment length polymorphism (RFLP-PCR) group, high-quality studies, and the population based (PB) and hospital based (HB) groups (Table 3).

### Sensitivity Analysis

**Fig 3:**
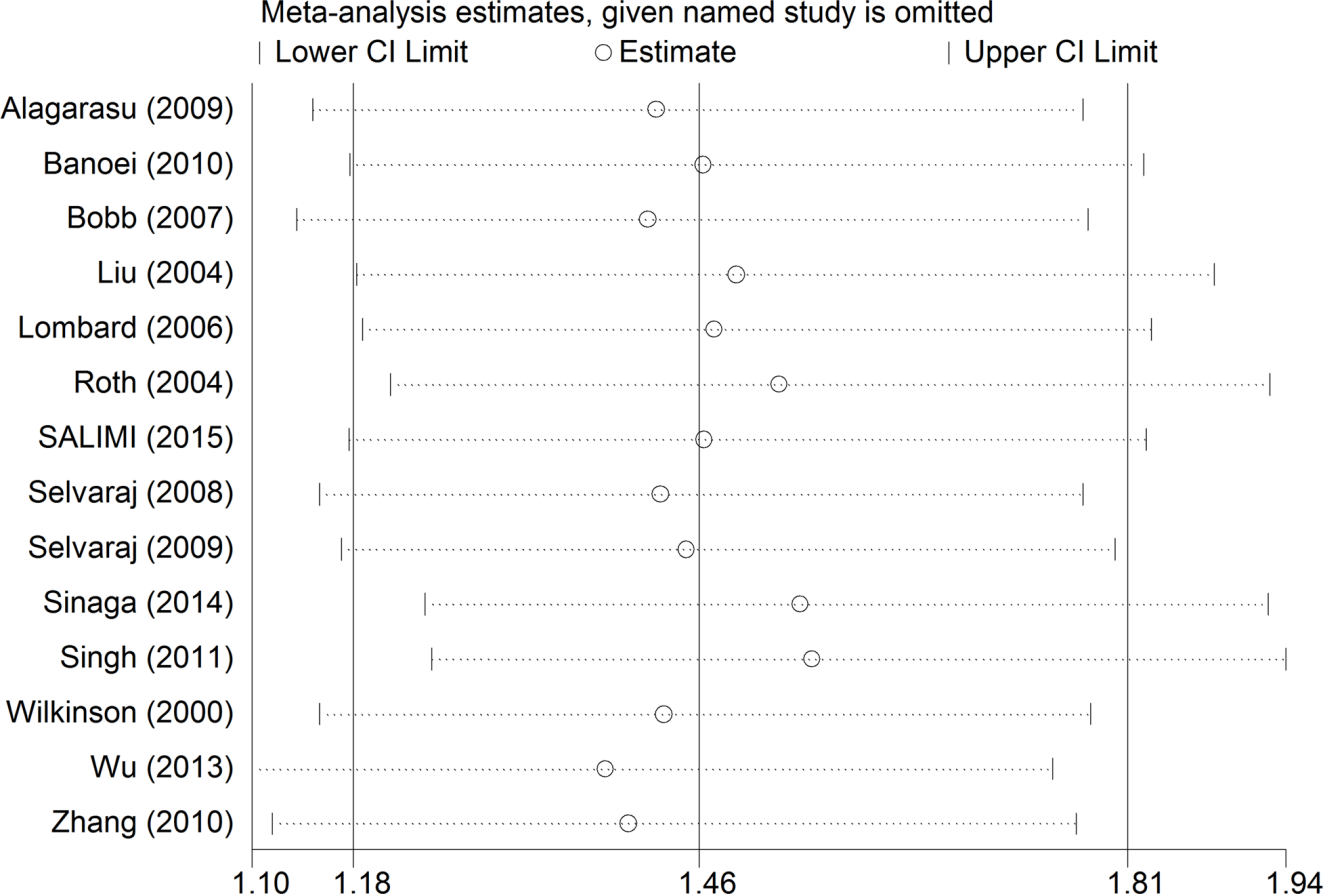
Sensitivity analysis for VDR *Fok*I Polymorphism and HIV-negative TB risk in recessive model. This figure shows the influence of individual studies on the summary OR. The middle vertical axis indicates the overall OR and the two vertical axes indicate its 95% CI. Every hollow round indicates the pooled OR when the left study is omitted in this meta-analysis. The two ends of every broken line represent the 95% CI.

Fig 3 shows the sensitivity analysis for VDR *Fok*I polymorphism and HIV-negative TB risk in recessive model. We first excluded the study of Selvaraj et al. [14], which was out of HWE, and the corresponding pooled ORs were not qualitatively altered (OR = 1.58, 95% CI = 1.27–1.92, P<0.001). Statistically similar results were obtained after sequentially excluding each study.

### Publication Bias

**Fig 4:**
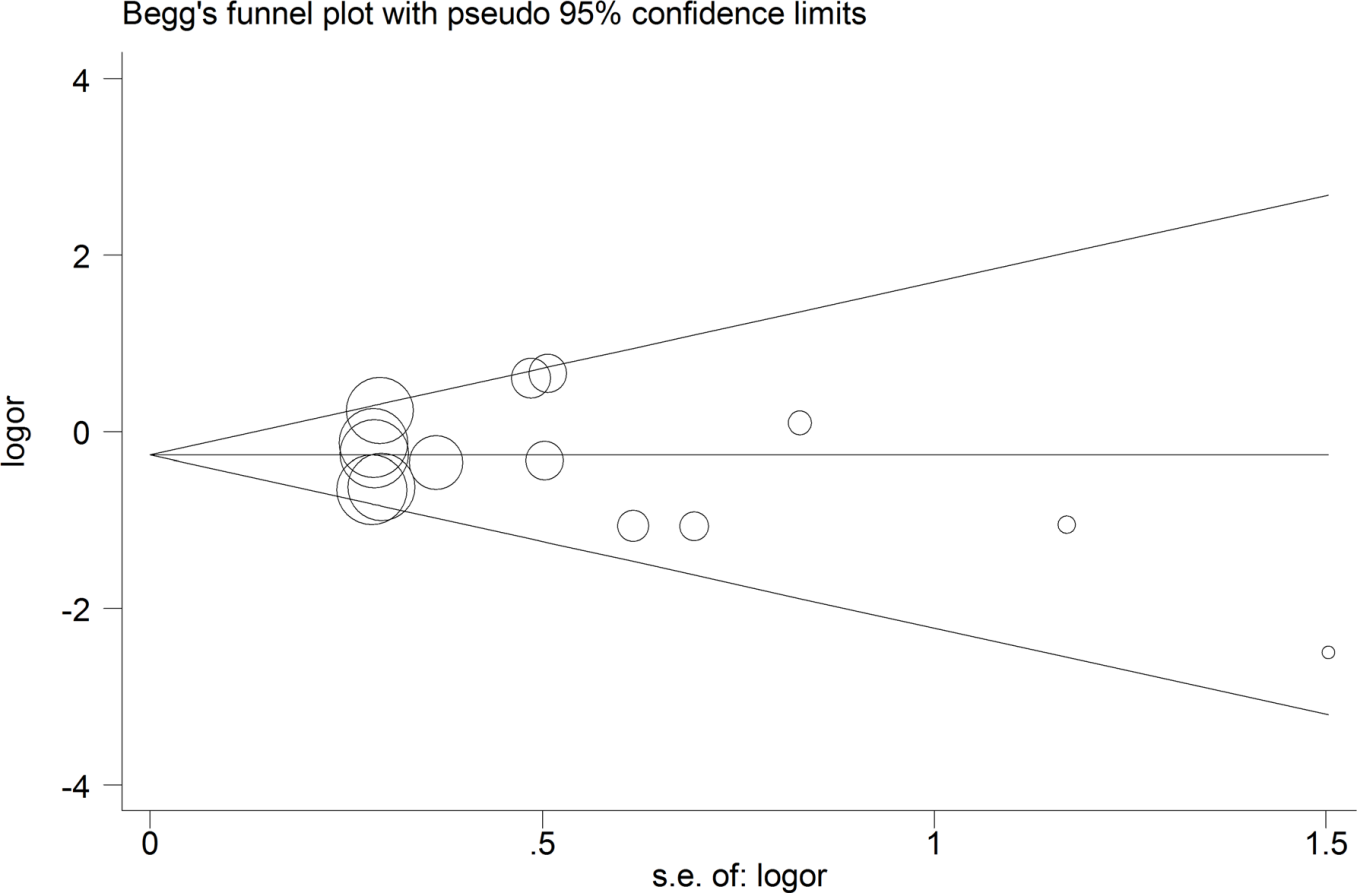
Begg’s funnel plot for contrast in overall analysis in recessive model. Each point represents a separate study for the indicated association. Size graph symbol by weights. *Log[OR]* natural logarithm of OR. Horizontal line mean effect size.

Fig 4 shows the Begg’s funnel plot in the recessive model. No significant publication bias was detected in the overall population. The statistical results of the Egger’s test also provided evidence of funnel plot symmetry (*P*_Egger’s test_ = 0.682, 95% CI = –2.078–1.406).

## Discussion

The identification of host genetic factors, such as human leucocyte antigens (HLA), cytokines, and receptors, have been studied extensively to determine susceptibility to TB [10, 31, 32]. However, the results of these studies are different even for the same gene polymorphisms across populations. These inconsistent results might be due to various factors, such as various racial factors, different genotyping methods, and the characteristics of the patients included, such as age and sex. In addition, studies from India and other parts of the world have shown that genetic susceptibility to TB is influenced by HIV infection [33, 34]. However, many previous studies have not excluded HIV-positive TB patients from case groups [35–37], which would bring significant selection bias in case-control studies. Therefore, we carried out a meta-analysis focusing on the association between VDR *Fok*I polymorphism and the risk of HIV-negative TB. Our results suggests that individuals with an ff genotype increased about 1.60-fold risk of TB compared with F carries (FF or fF genotype) in the HIV-negative population, and 1.82-fold in the Asian group. Moderate heterogeneity was found in this study.

VDR *Fok*I polymorphism increasing the risk of TB is biologically plausible. Vitamin D is an important immunoregulatory hormone; 1,25(OH)2D3, the active form of vitamin D, modulates the production of monocytes, lymphocytes, and several interleukins and other cytokines, as well as various oncogenes and transcription factors via VDR. Upon binding to 1,25(OH)2D3, the VDR complex moves into the nucleus, where it regulates the expression of genes [38]. The activated VDR also plays a role in regulating the adaptive immune system by inhibiting lymphocyte proliferation and reducing the production of pro-inflammatory cytokines to prevent excessive responses [39]. A significant interaction between vitamin D status and VDR gene polymorphisms was also observed among Gujarati Asians in West London [40]. The significant association between low vitamin D levels and susceptibility to TB infection has also been found [41]. These studies suggested that VDR gene polymorphisms can influence the function of vitamin D and thus contribute to the susceptibility to TB infection.

As we know, gene polymorphisms are complicated and fluctuating, mainly due to various races and regions. Moreover, the burden of TB is highest in Asia and Africa geographically [1]. Therefore, although significant heterogeneity was not found in this meta-analysis, we still performed a subgroup analysis by ethnicity to best understand the race-specific effects on the association between VDR *Fok*I polymorphism and the risk of HIV-negative TB. As a result, a significant association was found in the Asian group when the ff genotype was compared with the FF and fF genotypes (OR = 1.82, 95% CI = 1.42–2.33), but not in the African and South American groups. However, there were only two studies focusing on African populations and one focusing on the South American population. In addition, we noticed that most of these studies from Asia were performed on Indian and Chinese populations. Considering that genetic background may be distinct among different populations, further studies on this topic in different ethnicities are expected to be conducted to strengthen our results.

Heterogeneity is the most common problem when explaining the results of a meta-analysis. Moderate heterogeneity was found in this study. However, heterogeneity was significantly reduced compared with previous meta-analyses [20, 21]. These results not only could confirm HIV infection status as the main source of heterogeneity in previous meta-analyses, but are also the reason why we conducted this study. Moderate heterogeneity in this meta-analysis was reasonable for various racial and different genotyping methods and the characteristics of the patients included, such as age and sex. We also carried out sensitivity analysis, and the corresponding pooled OR value did not differ significantly from that of the overall meta-analysis. Moreover, a Begg’s funnel plot and an Egger’s test showed no publication bias. These results indicate that our results are reliable.

The strengths of this study include focusing on HIV-negative TB patients to avoid selection bias, and the heterogeneity was significantly reduced compared with previous meta-analyses. We also used the best genetic model to avoid the problem of multiple comparisons. Therefore, although our results were similar to those of previous meta-analyses [21, 42], we think that our results are more credible than previous studies and are closed to the true relationship between *Fok*I polymorphism and TB. The main limitation of this study is that a more precise analysis could not be conducted of individual information, including other covariates such as age and sex, due to a lack in the original data of the reviewed studies. In addition, most of the case—control studies were conducted in Asians; thus, our results may be applicable only to this ethnic group. Finally, this study could not address gene—gene and gene—environment interactions.

## Conclusion

In summary, our meta-analysis suggested that VDR *Fok*I polymorphism contributes to increasing the risk of TB in HIV-negative individuals, especially in the Asian region. Further studies on this topic in other races are expected to be conducted in future.

## Supporting Information

S1 PRISMA Checklist PRISMA 2009 Checklist. (DOC)

S1 Table Meta-analysis-on-genetic-association-studies-form. (DOCX)

S2 Table Full-text articles excluded with reasons. (DOCX)
