# Supplementary material for: Vitamin D Receptor Gene FOKI Polymorphism Contributes to Increasing the Risk of HIV-Negative Tuberculosis: Evidence from a Meta-Analysis
Source: PLoS One. 2015 Oct 20;10(10):e0140634. doi: 10.1371/journal.pone.0140634 (PMC4618110; doi:10.1371/journal.pone.0140634)
Supplement: S2 Table — (DOCX) [file pone.0140634.s003.docx]

**Full-text articles excluded, with reasons:**

Could not get sufficient data from author: [1, 2]

Not VDR gene FOKI: [3-6]

No data for HIV infection status: [7-20]

Mixed (HIV- and HIV+) patients [21-23]

Meeting Abstracts: [24]

Non-tuberculous mycobacterial lung disease [25]

1. Arji N, Busson M, Iraqi G, Bourkadi JE, Benjouad A, Bouayad A, Mariaselvam C, Salah S, Fortier C, Amokrane K *et al*: **Genetic diversity of TLR2, TLR4, and VDR loci and pulmonary tuberculosis in Moroccan patients**. *Journal of infection in developing countries* 2014, **8**(4):430-440.

2. Motsinger-Reif AA, Antas PRZ, Oki NO, Levy S, Holland SM, Sterling TR: **Polymorphisms in IL-1(beta), vitamin D receptor Fok1, and Toll-like receptor 2 are associated with extrapulmonary tuberculosis**. *BMC medical genetics* 2010, **11**(1).

3. Bellamy R: **Identifying genetic susceptibility factors for tuberculosis in Africans: a combined approach using a candidate gene study and a genome-wide screen**. *Clinical science (London, England : 1979)* 2000, **98**(3):245-250.

4. Bellamy R, Ruwende C, Corrah T, McAdam KPWJ, Thursz M, Whittle HC, Hill AVS: **Tuberculosis and chronic hepatitis B virus infection in Africans and variation in the vitamin D receptor gene**. *Journal of Infectious Diseases* 1999, **179**(3):721-724.

5. Delgado JC, Baena A, Thim S, Goldfeld AE: **Ethnic-specific genetic associations with pulmonary tuberculosis**. *The Journal of infectious diseases* 2002, **186**(10):1463-1468.

6. Sudarto, Ahmad Z, Arrahmanda K, Ergan D, Rasyid A, Yuwono: **Effect of Taqi vitamin D receptor gene polymorphism on the incidence of pulmonary tuberculosis**. *Respirology* 2011, **16**:78.

7. Kang TJ, Jin SH, Yeum CE, Lee SB, Kim CH, Lee SH, Kim KH, Shin ES, Chae GT: **Vitamin D Receptor Gene TaqI, BsmI and FokI Polymorphisms in Korean Patients with Tuberculosis**. *Immune network* 2011, **11**(5):253-257.

8. Marashian SM, Farnia P, Seyf S, Anoosheh S, Velayati AA: **Evaluating the role of vitamin D receptor polymorphisms on susceptibility to tuberculosis among iranian patients: a case-control study**. *Tuberkuloz ve toraks* 2010, **58**(2):147-153.

9. Varahram M, Farnia P, Anoosheh S, Kazampour M, Merza M, Saeif S, Masjedi MR, Velayati AA: **The VDR and TNF-(alpha) gene polymorphisms in Iranian tuberculosis patients: The study on host susceptibility**. *Iranian Journal of Clinical Infectious Diseases* 2009, **4**(4):207-213.

10. Ates O, Dolek B, Dalyan L, Musellim B, Ongen G, Topal-Sarikaya A: **The association between BsmI variant of vitamin D receptor gene and susceptibility to tuberculosis**. *Mol Biol Rep* 2011, **38**(4):2633-2636.

11. Joshi L, Ponnana M, Penmetsa SR, Nallari P, Valluri V, Gaddam S: **Serum vitamin D levels and VDR polymorphisms (BsmI and FokI) in patients and their household contacts susceptible to tuberculosis**. *Scandinavian journal of immunology* 2014, **79**(2):113-119.

12. Mahmoud AA, Ali AHK: **Vitamin D receptor gene polymorphism and 25 hydroxy vitamin D levels in Egyptian patients with pulmonary tuberculosis**. *Egyptian Journal of Chest Diseases and Tuberculosis* 2014, **63**(3):651-655.

13. Merza M, Farnia P, Anoosheh S, Varahram M, Kazampour M, Pajand O, Saeif S, Mirsaeidi M, Masjedi MR, Velayati AA *et al*: **The NRAMPI, VDR and TNF-alpha gene polymorphisms in Iranian tuberculosis patients: the study on host susceptibility**. *The Brazilian journal of infectious diseases : an official publication of the Brazilian Society of Infectious Diseases* 2009, **13**(4):252-256.

14. Rashedi J, Asgharzadeh M, Moaddab SR, Sahebi L, Khalili M, Mazani M, Abdolalizadeh J: **Vitamin d receptor gene polymorphism and vitamin d plasma concentration: correlation with susceptibility to tuberculosis**. *Advanced pharmaceutical bulletin* 2014, **4**(Suppl 2):607-611.

15. Rathored J, Sharma SK, Singh B, Banavaliker JN, Sreenivas V, Srivastava AK, Mohan A, Sachan A, Harinarayan CV, Goswami R: **Risk and outcome of multidrug-resistant tuberculosis: vitamin D receptor polymorphisms and serum 25(OH)D**. *The international journal of tuberculosis and lung disease : the official journal of the International Union against Tuberculosis and Lung Disease* 2012, **16**(11):1522-1528.

16. Selvaraj P, Narayanan PR, Reetha AM: **Association of vitamin D receptor genotypes with the susceptibility to pulmonary tuberculosis in female patients and resistance in female contacts**. *Indian Journal of Medical Research* 2000, **111**(MAY):172-179.

17. Selvaraj P, Chandra G, Kurian SM, Reetha A, Narayanan P: **Association of vitamin D receptor gene variants of BsmI, ApaI and FokI polymorphisms with susceptibility or resistance to pulmonary tuberculosis**. *Current Science* 2003, **84**(12):1564-1563.

18. Selvaraj P, Kurian SM, Chandra G, Reetha AM, Charles N, Narayanan PR: **Vitamin D receptor gene variants of BsmI, ApaI, TaqI, and FokI polymorphisms in spinal tuberculosis**. *Clin Genet* 2004, **65**(1):73-76.

19. Sharma PR, Singh S, Jena M, Mishra G, Prakash R, Das PK, Bamezai RNK, Tiwari PK: **Coding and non-coding polymorphisms in VDR gene and susceptibility to pulmonary tuberculosis in tribes, castes and Muslims of Central India**. *Infection, Genetics and Evolution* 2011, **11**(6):1456-1461.

20. Wilbur AK, Salter Kubatko L, Hurtado AM, Hill KR, Stone AC: **Vitamin D receptor gene polymorphisms and susceptibility M. tuberculosis in Native Paraguayans**. *Tuberculosis* 2007, **87**(4):329-337.

21. Bornman L, Campbell SJ, Fielding K, Bah B, Sillah J, Gustafson P, Manneh K, Lisse I, Allen A, Sirugo G *et al*: **Vitamin D receptor polymorphisms and susceptibility to tuberculosis in West Africa: A case-control and family study**. *Journal of Infectious Diseases* 2004, **190**(9):1631-1641.

22. Olesen R, Wejse C, Velez DR, Bisseye C, Sodemann M, Aaby P, Rabna P, Worwui A, Chapman H, Diatta M *et al*: **DC-SIGN (CD209), pentraxin 3 and vitamin D receptor gene variants associate with pulmonary tuberculosis risk in West Africans**. *Genes Immun* 2007, **8**(6):456-467.

23. Soborg C, Andersen AB, Range N, Malenganisho W, Friis H, Magnussen P, Temu MM, Changalucha J, Madsen HO, Garred P: **Influence of candidate susceptibility genes on tuberculosis in a high endemic region**. *Molecular immunology* 2007, **44**(9):2213-2220.

24. Kim JS, Kim Y, Moon H, Kim YK, Yoon HK, Lee SH, Kim S, Kim J, Kim CH: **Influence of vitamin d receptor polymorphism on tuberculosis among South Korean**. *American Journal of Respiratory and Critical Care Medicine* 2011, **183**(1).

25. Park S, Kim EJ, Lee SH, Suh GY, Chung MP, Kim H, Kwon OJ, Koh WJ: **Vitamin D-receptor polymorphisms and non-tuberculous mycobacterial lung disease in Korean patients**. *International Journal of Tuberculosis and Lung Disease* 2008, **12**(6):698-700.
